# Effectiveness of an Interdisciplinary Program Performed on Obese People Regarding Nutritional Habits and Metabolic Comorbidity: A Randomized Controlled Clinical Trial

**DOI:** 10.3390/ijerph17010336

**Published:** 2020-01-03

**Authors:** Virginia Esperanza Fernández-Ruiz, Antonio Jesús Ramos-Morcillo, María Solé-Agustí, José Antonio Paniagua-Urbano, David Armero-Barranco

**Affiliations:** 1 Nutrition Department, Hospital Clínico Universitario Virgen de la Arrixaca, 30120 El Palmar, Spain; virginiaesperanza.fernandez@um.es; 2 Department of Nursing, Faculty of Nursing, University of Murcia, 30100 Espinardo, Spain; darmero@um.es; 3 Murcian Health Service, 30008 Murcia, Spain; mariasole@hotmail.com (M.S.-A.); joseantonio.paniagua@um.es (J.A.P.-U.)

**Keywords:** nutrition, obesity, nurse, physical activity, program evaluation, BMI, randomized controlled clinical trial, metabolic diseases, individual nutrition education, group nutrition education

## Abstract

Obesity is an important public health problem. The combined use of different therapies performed by an interdisciplinary group can improve the management of this health issue. The main goal of this research is to determine the effectiveness of a multidisciplinary program based on healthy eating, exercise, cognitive-behavioral therapy, and health education in improving metabolic comorbidity, Body Mass Index (BMI), and nutritional habits among obese adults, at short (12 months) and long term (24 months). A randomized controlled clinical trial was conducted at a community care center between February 2014 and February 2016. A random sampling was done (299), total population (3262). A sample of 74 subjects diagnosed with obesity (experimental group, *n* = 37 and control group, *n* = 37) was conducted. Inclusion criteria: obese people (BMI: >30 kg/m^2^) with metabolic comorbidity and bad nutritional habits. Exclusion criteria: other comorbidities. A 12-month interdisciplinary program (with pre-test, 12 months and 24 months of follow-up) was applied. Intervention is based on healthy eating, exercise, and cognitive behavioral therapy. The intervention had a positive effect on nutritional habits (F2;144 = 115.305; *p* < 0.001). The experimental group increased fruit and vegetable intake (F2;144 = 39.604, *p* < 0.001), as well as fortified foods (F2;144 = 10,076, *p* < 0.001) and reduced fats, oils, and sweets F2;144 = 24,086, *p* < 0.001). In the experimental group, a BMI reduction of 2.6 to 24 months was observed. At follow-up, no participant had inadequate nutritional habits, compared to 35.1% of the control group (χ22 = 33,398; *p* < 0.001). There was also a positive response of metabolic comorbidities in the intervention group. The interdisciplinary program improved all participants’ metabolic parameters, BMI, and nutritional habits while maintaining the long-term effects (24 months).

## 1. Introduction

Obesity is a multifactorial disease that has been growing in the past few years despite the governmental efforts to stop and prevent it. The global prevalence of obesity and overweight has doubled since 1980, resulting in one third of the world population being obese or suffering overweight nowadays [1]. In Spain, in 2016, the prevalence of overweight in adult population was 39.5% and non-morbid obesity 19.5% [2]. This has been accompanied by a dramatic rise in type 2 diabetes, cardiovascular problems, and some kinds of cancer [3]. The numbers of metabolic comorbidity associating with these conditions (obesity and overweight) oscillate between 10% and 84%, depending on the pathologies involved in it [4,5].

There is a clear relationship between lifestyles and overweight and obesity [6]. Different types of diets have been described for the control of obesity and its comorbidity [7]. The Mediterranean diet is one of the most recommended for cardiovascular protection [8,9,10]. A study focused solely on a dietary intervention based on the Mediterranean diet on 7447 subjects found a reduction of 30% in cardiovascular risk [11]. Physical activity alone is not capable of achieving large weight losses, usually less than 5 kilos [12], although it does achieve notable benefits in other cardiovascular parameters such as diastolic blood pressure or triglycerides. However, research has shown that in the short term, lifestyle modification (diet and exercise) reduces obesity by obtaining cardiovascular benefits [13,14].

Behavioral modification through cognitive behavioral therapy for people with metabolic syndrome has demonstrated cardiovascular benefits, greater when it is a complement to other interventions [15,16].

The community context is an ideal place to implement interventions to promote physical activity, reduce sedentary lifestyles, and promote a healthy diet [17]. However, long-term results are limited. Current evidence argues that programs based on a single intervention encounter time limitations. In turn, dietary interventions, physical activity promotion, and individual behavioral therapies achieve modest reductions in weight and cardiovascular risk factors. However, weight control behavioral programs along with diet and physical activity are more effective [18].

Therefore, multicomponent and interdisciplinary therapeutic strategies are recommended [19,20,21,22]. Recent research suggests that if the leadership needed to manage these interventions is exercised by the nursing team, the effectiveness of these programs could be increased [23].

The aim of this study was to determine the effectiveness of a multidisciplinary program based on healthy eating, exercise, cognitive-behavioral therapy, and health education in improving metabolic comorbidity, body mass index (BMI), and nutritional habits among obese adults, at short (12 months) and long term (24 months).

## 2. Materials and Methods

### 2.1. Study Design

A randomized controlled trial was conducted with random allocation to the experimental (EG) or control group (CG) (independent measure). A 12-month intervention was conducted in EG (from February 2014 until February 2015), unlike participants in CG, who maintained the standard health checks included in the Community Care Program of the Public Health Service of Murcia (PHSM) (Spain) [24]. The effectiveness of the intervention was evaluated through the analysis of metabolic comorbidity (biochemical parameters), BMI, and nutritional habits examined before, during (12 months), and after the intervention on February 2016 (24 months).

### 2.2. Sample Size

Based on power calculations, 37 subjects were needed in each group (adjusted for 10% drop-out) to detect a difference of 0.4 BMI, assuming 0.34 standard deviation, with 80% power at the 9-month follow-up between the intervention and control group.

### 2.3. Setting, Recruitment, and Selection

This study was carried out in a community care center in the city of Murcia (Spain). The criteria for participation in the study was adults with BMI >30.0 kg/m^2^ and not started any treatment for obesity. Recruitment followed a standardized protocol, and patients who attended their physicians at primary care and met the participation criteria were asked to participate in the study.

**Figure 1:**
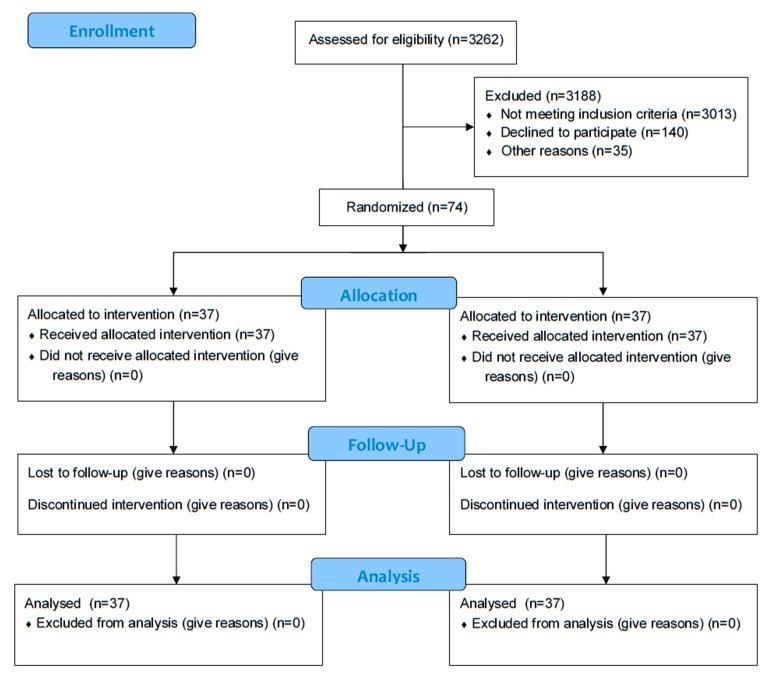
Participant flow diagram.

The health center served a population of 3262 people, but 3013 did not match the inclusion criteria. Of the 249 subjects who met the BMI criteria, 140 declined participation, and 35 were excluded because of different reasons that could interfere with the research (depression, cancer, fibromyalgia, and others). Finally, 74 people who met the condition of having a BMI superior than 30 agreed to participate in the program and signed the informed consent (Figure 1).

Each participant was randomly assigned to either the intervention or control group using a random-number table generated by a researcher with no involvement in the clinical trial and opaque envelopes were used to ensure concealment.

### 2.4. Intervention

#### 2.4.1. The Interdisciplinary Intervention against Obesity

The EG underwent an interdisciplinary program, with a comprehensive approach for the treatment of obesity for 12 months with a re-evaluation 12 months later. Nurses are responsible for establishing the objectives of the intervention, leading the interdisciplinary team which included, in addition to the nurse, a physician, a nutritionist, a psychologist, and a monitor of physical activity (MPA). The ongoing evaluation of the project, with a monthly meeting by all professionals was established and lead by the nurses. The services of the nutritionist, psychologist, and MPA were provided by the town hall.

In addition to the supervision tasks, the nurse carried out the following activities: development of health education contents, anthropometric controls, administration of food habits surveys, and management of the follow-up of analytical controls. To implement health education, by maintaining a monthly session of 60 min for educational treatment of metabolic syndrome and cardiovascular risk, modification of unhealthy dietary habits, and selection and preparation of healthy menus (12 sessions in total). The activities conducted by the MPA professional consisted of four weekly sessions of physical activity for forty minutes. It began with stretching exercises (ten minutes) followed by thirty minutes of moderate aerobic work for all ages (20 min of treadmill walking fast or light running) with a rest period at the end (208 total sessions). Psychologists conducted a monthly cognitive behavioral therapy session, sixty minutes, based on techniques of psychoeducation (motivation), cognitive restructuring, problem solving (self-efficacy), and skills training, (12 sessions in total). The physician and nutritionist conducted the clinical and nutritional evaluation, to monitor drug-nutrient interactions and monitor any imbalance or adverse reactions. A total of 12 sessions were conducted (1 per month). After, the energy needs and nutritional assessment were calculated using the formula of Harris and Benedict [25]. Both professionals individually instructed on dietary management [25]. The physician evaluated the analytical control, supporting part of the health education and carrying out the habitual patient follow-up.

#### 2.4.2. Control

The control group received the usual care offered by the Murcian Health Service [24]. Such care was offered when the person had cardiovascular disease, arterial hypertension, diabetes mellitus, hypercholesterolemia, or obesity. On the part of the nurse, and depending on the clinical evolution of the patient, more or less intense follow-ups were carried out. A check-up was usually carried out every 6 months in a nurse’s office if there was a good control, every 3 months if the control was moderate, and as many times as necessary in case of bad control. The interventions carried out were: health education, control of constants. The physician performed weight measurement and analytical control. In the primary care center, there was no nutritionist, psychologist, or MPA.

The only activity that the research team performed on patients assigned to the control group was the anthropometric and clinical evaluation, conducted in the primary care center, at the aforementioned time points. Similarly, during the post-intervention year, the research team had no contact with the participants. Patients were contacted again one year after completion of the intervention, conducting the last anthropometric and clinical evaluation.

### 2.5. Outcomes Measures

All measurements were performed at three time moments. The first two, aimed at measuring the effectiveness of the intervention in the medium term: before starting the intervention and at 12 months (medium term, when the intervention ends). In order to know the long-term effect of this intervention, a last measurement was made one year after it was concluded (24 months).

Control variables: Variables that were considered to be of interest to control their confounding effect were evaluated. Socio-demographic data: sex, age and toxic habits: smoking, alcohol consumption.

Anthropometric data. The BMI was determined. BMI was calculated as the ratio of weight in kilograms to height in meters squared [26]. Weight and height were measured on a scale calibrated with a portable stadiometer.

Comorbidity data. The biochemical parameters that determine the main metabolic diseases were evaluated: diabetes mellitus (DM) values of basal glycemia (BG) and glycosylated hemoglobin (HbA1c); dyslipidemia values of total cholesterol (TC) and triglycerides (TG); hepatic disorder (HD) values of gamma glutamyltranspeptidase (GGT), glutamicopyruvic transaminase (GPT), glutamate-oxalacetic transaminase (GOT), and total bilirubin (TB). Blood samples were extracted after a night fast of 10–12 h. We analyzed hypertension (HTN) through systolic blood pressure (SBP) and diastolic blood pressure (DBP). For the determination of these comorbidities, international standards were followed and they were diagnosed by the team physician. They were performed in the laboratory service of a university hospital that met the standards of quality and accreditation.

Evaluation of nutritional habits. To evaluate the quality of the participants’ nutritional habits, two questionnaires were used: one of nutritional habits and another of food frequency (Food Consumption Frequency Questionnaire, FCFQ).

The questionnaire on nutritional habits is based on the recommendations of the Spanish Society of Community Nutrition [27]. This questionnaire has been used in several studies on eating behavior [28,29]. It consists of 17 items that measure various aspects of eating behavior quality: food contents, time and place of intake, duration. The items are multiple choice, except for the first. They consist of three response categories: A, which indicates the healthiest habit, 3 points, B, less healthy habit, 2 points, C, unhealthy habit, 0 points. The first item (number of meals per day), however, consists of 5 categories (the 5 usual intakes throughout the day). Three points are obtained if the options breakfast, lunch and dinner are marked, together with, as a minimum, another additional category. If the categories breakfast, lunch and dinner are marked, 2 points are obtained, and 0 points in any other variation.

The total score is composed of the sum of the score obtained in each item, the maximum score attainable is 51, which indicates the best nutritional habits, and the minimum is 0 which indicates that all nutritional habits are harmful to health. Simonelli [28] suggested two cut-off points (≥23, ≥31) that create 3 kinds of nutritional habits (balanced diet ≥ 31; quiet-unbalanced diet ≥ 23–< 31; unbalanced diet < 23).

The FCFQ evaluates the frequency of intake of 88 classes of foods and fortified foods (omega-3, vitamins A, C, and E, soy) representative of all food pyramid strata [28]. Food belongs to 7 food groups (FG): Group 1 (10 items): bread, cereals, rice, pasta, and legumes; Group 2 (13 items): meat, poultry, fish, and eggs; Group 3 (23 items): fruits and vegetables; Group 4 (13 items): milk, yogurt, and cheese; Group 5 (23 items): fats, oils, and sweets; Group 6 (3 items): water; Group 7 (3 items): fortified foods.

All questionnaires were self-administered and completed in the presence of the nurse, giving the possibility to clarify the doubts that may have arose, avoiding losses in the sample.

### 2.6. Statistical Analysis

Statistical analyses were performed with IBM SPSS^®^ statistical software, 22.0 (IBM Corp, Armonk, NY, USA). Intention-to-treat (ITT) analysis was carried out. A descriptive study of the variables was performed, calculating the mean, standard deviation, minimum, and maximum. The effectiveness of the intervention was analyzed by applying a partly repeated measures analysis of variance (ANOVA) (mixed-design analysis of variance), taking the group as between-subject factor and the time of measurement of the dependent variable as the within-subject factor. There was an effect in the intervention when the F interaction was statistically significant. In this case, to study the longitudinal development of the dependent variable within each group, an analysis of variance for repeated measures was applied. The evaluation of the effective size was performed using the statistical partial η^2^. In repeated measures, ANOVA pairs of time points were compared by the Tukey test. We used Bonferroni correction for multiple comparisons. The analysis of the association of the classification in the nutritional habits with the group in each one of the evaluations was carried out with the Chi-square test. A logistic binary regression for metabolic comorbidities was applied to predict metabolic comorbidities with dietary intake at the end of the intervention and follow-up as predictors. Statistical significance was set at *p* < 0.05.

Data collector and data analyst were unaware of the treatment the participants receive.

### 2.7. Ethical Considerations

The research was approved by the Ethics Committee of the University Hospital Reina Sofia (ID: JMG/012013) (30032015). Likewise, anonymity and confidentiality of data and information concerning patients was guaranteed, following the ethical principles of the Declaration of Helsinki. After showing the objectives of the study and asking the necessary questions, the participants completed a consent form.

## 3. Results

Nobody was withdrawn from the study in any of the groups during the follow-up (Figure 1). The socio-demographic variables and metabolic antecedents are presented in Table 1. The groups are balanced in age, gender, BMI, toxic habits, and metabolic comorbidity (DM, DLP, HTN, HD).

The mean difference of the BMI in the GC between the initial time and 12 months was −0.1 (BMI pretest: 34.3, SD = 4.5; BMI 12 m-34.2 SD = 4.2) and between the initial time and 24 months was 0.3 (BMI pretest: 34.3, SD = 4.5; BMI 24 m-34.6 SD = 4.1). The mean difference of the BMI in the EG between the initial time and 12 months was −2.6 (BMI pretest: 32.4, SD = 3.8; BMI 12 m 29.8 SD = 3.3) and between the initial time and 24 months was −2.7 (BMI pretest: 32.4, SD = 3.8; BMI 24 m-29.7, SD = 3.3). The results for BMI significantly differed, both between the groups (F = 16.87, *p* < 0.001) and in the interaction of intervention with time (F = 77.52, *p* < 0.001).

The analysis of the effects of the intervention on nutritional habits (Table 2) reveals that the participants who received the treatment had a positive evolution (F2;144 = 115.305; *p* < 0.001) at the end of the treatment (*p* < 0.001) and in follow-up compared to pretest and completion of treatment (*p* < 0.001). The CG kept the same score during the evaluations.

The analysis of the frequency of intake of bread, cereals, rice, pasta, and legumes (Food Group 1; FG1), meat, poultry, fish, and eggs (FG2), milk, yogurt, and cheese (FG4) and Water (FG6) shows that the intervention did not had an effect on these food groups.

Regarding the intake of fruits, vegetables (FG3), both groups were different in the pretest (see footnote FG3 in Table 2), with lower intake in EG. The analysis of the simple effects of the moment in each group shows that the participants who received the treatment had increased their intake (F2;144 = 39.604; *p* < 0.001) at 12 months and in the follow-up with respect to the pretest (*p* < 0.001). In CG, there are no changes in the consumption of fruits and vegetables (F2;144 = 2.044; *p* = 0.133).

The intake of fats, oils, and sweets (FG5) showed pretest differences, being lower in EG. After the intervention and in the follow-up, EG participants had decreased their intake (*p* < 0.001), showing a positive evolution (F2;144 = 24,095; *p* < 0.001). In CG, a higher frequency of fat, oil, and sweets (F2;144 = 17,363; *p* < 0.001) was observed in the follow-up compared to the pretest and the end of the treatment (*p* < 0.001).

The frequency of consumption of enriched foods showed a positive evolution in the EG (F2;144 = 10.064; *p* < 0.001), both at the end of treatment (*p* < 0.001) and at follow-up (*p* < 0.001) compared to the pretest. CG did not experience changes in the assessments performed (F2;144 = 0.905; *p* = 0.407).

**Figure 2:**
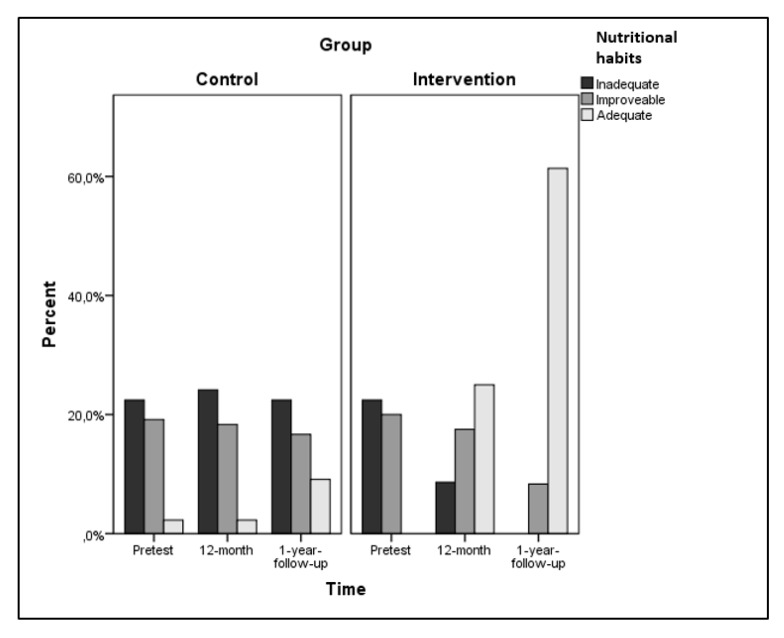
Classification of participants in intervention and control groups into nutritional habit categories.

Regarding the classification of participants in the categories of nutritional habits (Figure 2), in EG, 35.1% had inadequate nutritional habits in the pretest, 64.9% could be improved, and none had adequate habits. These figures were similar (χ22 = 1.021, *p* = 0.600) to CG (inadequate habits: 35.1%, could be improved: 62.2%, adequate: 2.7%). At 12 months, 13.5% of EG participants were classified with inadequate habits, 56.8% improved and 29.7% were adequate; while in CG, 37.8% had inadequate nutritional habits, 59.5% improved, and 2.7% adequate (χ22 = 12,620, *p* = 0.002). These results enhanced after the intervention (24 months), as none of the participants in EG remained in the group of inappropriate habits, 27% had improved habits and 73% had adequate ones; In CG, 35.1% had inadequate habits, 54.1% improved, and 10.8% adequate (χ22 = 33.398, *p* < 0.001).

We also found a positive response of the clinical and biochemical parameters that represent the comorbid pathologies of obesity in EG, with the exception of HTN which showed reduced numbers for both groups (Table 3). Thus, SBP improved both in CG (F2;144 = 20,727; *p* < 0.001) and in EG (F2;144 = 191,598; *p* < 0.001). In both groups, they decreased at 12 months, remaining stable at follow-up. However, SBP values were lower in EG at 12 months (F1;144 = 150.755, *p* < 0.001) and at follow-up (F1;144 = 173.434; *p* < 0.001). The same evolution was found in DBP, decreasing in CG (F2;144 = 53.102; *p* < 0.001) and in EG (F2;144 = 382.591; *p* < 0.001), being higher in EG at 12 months (F1;144 = 64.385, *p* < 0.001) and follow-up (F1;144 = 308.188, *p* < 0.001).

Regarding the parameters analyzed in relation to DLP, CT values increased in CG (F2;144 = 7.198; *p* = 0.001), being higher at follow-up than at 12 months (*p* = 0.001). In EG, there was a decrease (F2;144 = 166.476; *p* < 0.001) at 12 months and at follow-up (*p* < 0.001). The effect of the intervention on TG in EG was significant (F2;144 = 43.389; *p* < 0.001), with a decrease at the end of the intervention and at follow-up, compared to the pretest (*p* < 0.001), although the values at follow-up remained stable after completion. In CG, there was a variation (F2;144 = 4.971; *p* = 0.008), which in this case manifested itself in an increase in TG at follow-up regarding the completion of the intervention.

The evolution of DM indicators showed a positive response. Glucose did not change in CG (F2;144 = 2.788; *p* = 0.065). In EG, they decreased (F2;144 = 71.399; *p* < 0.001) at 12 months and at follow-up (*p* < 0.001), remaining stable after completion. HbA1c decreased in EG (F2;144 = 116.928; *p* < 0.001) at 12 months and at follow-up (*p* < 0.001), being equivalent in these last two evaluations. In CG, there was a variation of HbA1c (F2;144 = 3.323; *p* = 0.039), although in this case the values at follow-up were higher than at the end of the intervention (*p* = 0.043).

Parameters for hepatic impairment were analyzed. The values of GOT (F2;144 = 2.930, *p* = 0.057), GPT (F2;144 = 0.754, *p* = 0.472), and GGT (F2;144 = 1.360, *p* = 0.260) have not varied in CG. In EG, GOT (F2;144 = 9.510; *p* < 0.001), GPT (F2;144 = 19.820; *p* < 0.001), and GGT (F2;144 = 28,070; *p* < 0.001) decreased, with a decrease at 12 months and at follow-up (*p* < 0.001), remaining stable after completion of the intervention. Regarding TB values, there was no variation in CG (F2;144 = 0.767, *p* = 0.466). In EG, there was an increase at 12 months (*p* < 0.001), to return to the pre-intervention values at follow-up (F2;144 = 14,497; *p* < 0.001) (Table 3).

The binary logistic regression models for the prediction of metabolic comorbidity at the end of the intervention and during the follow-up from the frequency of food group (FG) intake show that, at the end of the program, the intake of fats, oils, and sweets (OR = 10.7, 95% CI: 2.5–45.5, *p* = 0.001), while ingestion of enriched foods is a protective factor (OR = 0.5, 95% CI: 0.3–0.9, *p* = 0.021). The remaining FGs are not significant predictors of metabolic comorbidity. At follow-up, fats, oils, and sweets (FG5) are maintained as a significant risk factor for metabolic comorbidity (OR = 72.6, 95% CI: 9.2–572.4, *p* = 0.001). On this occasion, the intake of meat, poultry, fish, and eggs (FG2) is a protective factor (OR = 0.06, 95% CI: 0.005–0.720, *p* = 0.001). The remaining predictors were not statistically significant.

## 4. Discussion

Scientific literature has shown for decades the individual and collective negative impact of obesity [30,31,32,33,34]. There is no debate that the only effective long-term programs should be interdisciplinary [35,36]. Some authors confirm how difficult is for subjects following their programs to maintain their improvements at long term [37], and a systematic review states that more research with follow-up for longer periods than 6 months are needed [38]. The maintenance of lost weight at long term is a generalized concern among researchers, therefore, in order to reach that goal, they associate different strategies (caloric restriction, increase of physical activity, etc.) [39], in addition of a multidisciplinary approach [35]. In this context, the methodology has been based on the association between a proper diet, exercise adapted to obese people, and behavioral therapy, together with an interdisciplinary approach including nursing leadership as a novelty. 

The results obtained in the medium and long term, both to correct nutritional habits and metabolic comorbidity are encouraging, attaining success rates at the end of the program and maintaining them after 12 months after the intervention. In this line, a 12-month program carried out on obese subjects demonstrated a significant association between weight loss with all biochemical and clinical parameters of metabolic comorbidities (HTN, DLP, and DM) (*p* < 0.001); EG participants who lost 5%–10% of body weight increased the odds of reduction of 0.5% HbA1c, a decrease of 5 mmHg in BPD, a decrease of 5 mmHg in SBP, and decrease of 40 mg in TG [40]. The DiRECT study following only 12 months, with dietary interventions performed by a nurse or dietitian (depending on availability) also shows favorable results in BMI (weight), HbA1c, DBP, and quality of life [26]. In a recent trial performed on overweight or obese adults with metabolic syndrome, the participants tried a Mediterranean diet with caloric restriction, together with physical exercise and behavioral support for 12 months. Results showed that those being part of the EG lost an average of 3.2 kg while participants in the CG only lost 0.7 kg (*p* < 0.002). The reduction on the resistance to insulin and the drop of HbA1c levels were bigger in individuals in the EG than in those from the CG (*p* < 0.05). EG members with prediabetes/diabetes improved significantly in their blood sugar level control and insulin sensitivity, together with triglycerides and HDL cholesterol levels in comparison with the CG group [41]. Berzigotti maintains the above and adds the positive correlation of the modification of habits in the hepatic parameters of obese subjects [42]. Our study confirms this correlation, EG subjects have shown an improvement in hepatic and TB enzymes at 12 months and at follow-up at 24 months, unlike CG which did not change their figures.

In reference to nutritional habits, Champagne observed that the increase in fruit and vegetable consumption was associated with significant weight loss; it was also found that the substitution of fat and carbohydrate intake for protein was associated with a significant decrease in weight [43]. Our research supports the findings, keeping in line with the results of the PREDIMED study [44]. The QUOVADIS study suggests that one of the keys to success in maintaining achievements in obese patients is that individuals acquire the capacity for autonomy without feeling the need for professional help [45]. Once the individual acquires the knowledge that will allow him to manage his own diet, there is still one more step as showed by their research [46]; the key for weight loss through diet is the maintenance of good habits in time, and is in this aspect where healthcare professionals become so important. Our program provides, not only a scheduled appropriate physical activity plan maintained for 12 months, but also dietary advice and cognitive-behavioral therapy offered by experts during those 12 months. We find these are key factors in the success of our program.

The experimental group had community support, as they were offered services by the town hall. There is now evidence to suggest that people with morbidities treated in primary care and with support from the community improve health outcomes [47].

Among the intrinsic limitations of the study is the fact that all subjects belong to the same community and blinding of the participants could not be possible. This could be related to a transfer of information from one group to another, altering the effects of the program. Despite this, the groups still have important differences in their results. This is a first approach for the analysis of the effectiveness of this type of intervention. Therefore, more studies with a multi-center character are required to control this possible bias by reducing the possibility of information exchange among the subjects under analysis.

## 5. Conclusions

In conclusion, the present clinical trial shows solid evidence regarding the effectiveness in the medium and long term in nutritional habits and metabolic comorbidity of subjects with obesity. This intervention has important effects on metabolic parameters such as SBP, DBP, TC, TG, glucose, Hb1c, GOT, GPT, GGT, and TB, as in the BMI. This research provides evidence that a multidisciplinary team supported by community resources and led by nurses is able to achieve significant improvements.

## Figures and Tables

**Table 1:** Age, gender, BMI, and clinical history prior to intervention.

	CG *n* = 37		EG *n* = 37		Test		
	M/N	SD/%	M/N	SD/%	Statistic	Df	*P*
Age (years)	62.8	8.9	59.4	9.1	1.646 ª	72	0.104
Gender (male)	18	48.6	19	51.4	0.000 ^b^	1	1.000
BMI (kg/m^2^)	34.3	4.5	32.4	3.8	1.979 ª	72	0.052
Smoking							
Non-smoker	26	70.3	20	54.1	2.177 ^b^	2	0.337
Former smoker	9	24.3	13	35.1			
Smoker	2	5.4	4	10.8			
Alcohol	22	59.5	21	56.8	0.000 ^b^	1	1.000
DM	23	62.2	16	43.2	1.952 ^b^	1	0.162
HTN	32	86.5	29	78.4	0.373 ^b^	1	0.541
DLP	21	56.8	19	51.4	0.054 ^b^	1	0.816
HD	1	2.7	1	2.7	0.000 ^b^	1	1.000

BMI: Body Mass Index. CG: control group. EG: experimental group. M: mean; SD: standard deviation; N: frequency; %: percentage; df: degrees of freedom; *p*: *p*-level. ^a^ T-test; ^b^ Chi-squared test. Diabetes mellitus (DM). Arterial hypertension (HTN). Dyslipidemia (DLP). Hepatic disease (HD).

**Table 2:** Effect of the intervention on the parameters of nutritional habits.

		Control *n* = 37		Intervention *n* = 37		Interaction	Moment	Group
		M	SD	M	SD	F_2;144_ *p* η^2^	F_2;144_ *p* η^2^	F_1;72_ *p* η^2^
Nutritional Habits ^a^	Pretest	28.5	5.3	28.5	6.2	52.029	63.928	28.183
	12-month	27.9	5.3	33.2	7.1	<0.001	<0.001	<0.001
	Follow-up	29.1	7.4	43.2	6.2	0.419	0.470	0.281
FG1 ^b^	Pretest	3.1	0.4	3.0	0.4	0.286	0.087	0.670
	12-month	3.1	0.3	3.0	0.3	0.752	0.917	0.416
	Follow-up	3.1	0.4	3.0	0.3	0.004	0.001	0.009
FG2 ^c^	Pretest	3.2	0.4	3.1	0.4	1.679	2.480	0.837
	12-month	3.2	0.4	3.1	0.4	0.190	0.087	0.363
	Follow-up	3.2	0.5	3.2	0.3	0.023	0.033	0.011
FG3 ^d^	Pretest	3.5	0.6	3.0	0.5	26.034	15.588	0.254
	12-month	3.5	0.6	3.5	0.5	<0.001	<0.001	0.616
	Follow-up	3.4	0.5	3.6	0.4	0.266	0.178	0.004
FG4 ^e^	Pretest	2.6	0.6	2.4	0.5	2.280	0.233	1.713
	12-month	2.5	0.5	2.5	0.4	0.106	0.792	0.195
	Follow-up	2.6	0.5	2.5	0.4	0.031	0.003	0.023
FG5 ^f^	Pretest	2.6	0.6	2.3	0.5	35.440	5.845	25.981
	12-month	2.6	0.7	2.0	0.4	<0.001	0.004	<0.001
	Follow-up	2.9	0.6	1.9	0.3	0.330	0.075	0.265
FG6 ^g^	Pretest	2.5	0.8	2.5	0.8	1.641	0.061	0.027
	12-month	2.5	0.9	2.5	0.6	0.197	0.941	0.870
	Follow-up	2.4	0.9	2.5	0.6	0.022	0.001	0.000
FG7 ^h^	Pretest	1.7	0.9	1.7	1.1	8.114	2.874	4.000
	12-month	1.6	1.0	2.2	1.4	<0.001	0.060	0.049
	Follow-up	1.5	1.1	2.4	1.4	0.101	0.038	0.053

CG: control group. EG: experimental group. FG; food group. M: mean; SD: standard deviation; Fdf1; df2: Snedecor’s F statistic; *p*: *p*-level; η2: partial eta squared. Pretest tests: ^a^ nutritional habits: t72 = 0.000; *p* = 1.000; ^b^ FG1: t72 = 0.903; *p* = 0.370; ^c^ FG2: t72 = 1.423; *p* = 0.159; ^d^ FG3: t72 = 3.461; *p* = 0.001; ^e^ FG4: t72 = 1.811; *p* = 0.074; ^f^ FG5: t72 = 2.767; *p* = 0.007; ^g^ FG6: t72 = 0.448; *p* = 0.656; ^h^ FG7: t72 = −0.193; *p* = 0.848.

**Table 3:** Effects of intervention on metabolic parameters.

		CG *n* = 37		EG *n* = 37		Interaction	Moment	Group
		M	SD	M	SD	F_3;216_ *p* η^2^	F_3;216_ *p* η^2^	F_1;72_ *p* η^2^
SBP ^a^ (mmHg)	Pretest	152.3	11.8	150.6	12.9	43.948	168.376	47.033
	12-Month	144.1	7.3	127.3	7.0	<0.001	<0.001	<0.001
	Follow-up	145.5	6.6	127.6	5.8	0.379	0.700	0.395
DBP ^b^ (mmHg)	Pretest	87.3	6.8	87.6	5.8	87.778	347.916	39.925
	12-Month	80.3	4.7	71.5	4.2	<0.001	<0.001	<0.001
	Follow-up	83.0	5.3	70.9	3.9	0.549	0.829	0.357
TC ^c^ (mg/dL)	Pretest	214.5	26.4	217.6	40.4	94.240	79.434	46.577
	12-Month	207.4	32.2	157.2	24.0	<0.001	<0.001	<0.001
	Follow-up	223.4	33.3	145.9	19.2	0.567	0.525	0.393
TG ^d^ (mg/dL)	Pretest	147.5	84.4	171.5	81.9	30.066	18.293	2.009
	12-Month	143.2	62.7	115.5	36.0	<0.001	<0.001	0.161
	Follow-up	164.9	69.7	110.3	28.9	0.295	0.203	0.027
Glucose ^e^ (mg/dL)	Pretest	139.1	33.4	132.9	36.0	27.584	46.603	40.406
	12-Month	129.1	27.2	88.4	9.7	<0.001	<0.001	<0.001
	Follow-up	136.6	31.4	86.8	6.8	0.277	0.393	0.359
HbA1c ^f^ (mg/dL)	Pretest	7.2	1.1	6.7	1.2	54.957	65.294	64.781
	12-Month	7.0	1.1	5.1	0.3	<0.001	<0.001	<0.001
	Follow-up	7.3	1.1	5.1	0.2	0.433	0.476	0.474
GOT ^g^ (U/L)	Pretest	22.7	9.7	23.0	10.7	8.248	4.192	4.565
	12-Month	22.0	6.5	19.1	2.8	<0.001	0.017	0.036
	Follow-up	24.6	6.1	18.5	2.1	0.103	0.055	0.060
GPT ^h^ (U/L)	Pretest	24.8	13.8	28.5	18.3	9.394	11.180	1.253
	12-Month	23.1	8.4	19.6	5.9	<0.001	<0.001	0.267
	Follow-up	25.1	6.7	18.1	2.6	0.115	0.134	0.017
GGT ^i^ (U/L)	Pretest	25.2	14.0	30.8	19.7	16.587	12.844	1.796
	12-Month	24.4	11.3	19.0	4.5	<0.001	<0.001	0.184
	Follow-up	27.4	11.2	17.9	1.7	0.187	0.151	0.024
TB ^j^ (mg/dL)	Pretest	0.5	0.2	0.5	0.2	20.822	5.964	1.104
	12-Month	0.5	0.2	0.6	0.3	<0.001	0.003	0.297
	Follow-up	0.5	0.2	0.5	0.1	0.224	0.076	0.015

CG: control group. EG: experimental group. M: mean; SD: standard deviation; Fdf1; df2: Snedecor’s F statistic; *p*: *p*-level; η2: partial eta squared. Pretest tests: ^a^ SBP: t72 = 0.603; p = 0.549; ^b^ DBP: t72 = −0.238; *p* = 0.812; ^c^ TC: t72 = −0.402; *p* = 0.689; ^d^ TG: t72 = −1.244; *p* = 0.217; ^e^ Glucose: t72 = 0.764; *p* = 0.447; ^f^ HbA1c: t72 = 1.897; *p* = 0.062; ^g^ GOT: t72 = −0.137; *p* = 0.892; ^h^ GPT: t72 = −0.997; *p* = 0.322; ^i^ GGT: t72 = −1.393; *p* = 0.168; ^j^ t72 = −1.356; *p* = 0.179.
